# The perspectives of women on sustainable fashion consumption: Comparative study of university teachers and students

**DOI:** 10.1371/journal.pone.0314532

**Published:** 2025-02-14

**Authors:** Živilė Stankevičiūtė, Ieva Jarmalavičiūtė

**Affiliations:** Kaunas University of Technology, School of Economics and Business, Kaunas, Lithuania; Coventry University, UNITED KINGDOM OF GREAT BRITAIN AND NORTHERN IRELAND

## Abstract

In the recent decade, fashion industry has been receiving extensive criticisms for having a significant negative impact on the environment and people. As the counterpoint of rapid pace and cheap prices which trigger overconsumption, sustainable fashion started turning into a new trend and demand for business. As the interest of consumers in sustainability continues to grow, the literature started analysing what drives or hinders them from sustainable consumption. However, acknowledging the gender differences, there is a gap in literature regarding sustainable fashion consumption from the perspective of female consumers. The purpose of this paper is to reveal the sustainable fashion consumption of females, comparing the clusters of university teachers and students. In doing this, a qualitative research was conducted, with 24 semi-structured interviews in total. The findings revealed a huge contradiction. Although females are aware of sustainable fashion elements, at the same time, they, especially students, do not consider sustainability as a primary motivation for their behaviour. As such, the gap between attitude and behaviour was revealed. Although teachers try to find the good ratio between quality and price, for students, the price usually wins when it comes to purchasing clothes. The representatives from both clusters do shopping from fast fashion retailers; however, students do it much more often and intensively in comparison to teachers. The teachers mainly use swapping and redesigning as sustainable fashion consumption practices. In case of students, second-hand shops and Vinted were prevailing. Among the drivers leading to sustainable consumption, awareness, uniqueness and personal style, and item life-cycle extension were mentioned. Regarding the barriers, high price and low income, personal restrictions, and lack of information and knowledge were mainly emphasised. The findings could be considered to initiate changes, which could enable closing the attitude-behaviour gap.

## Introduction

The fashion industry is a global business of 1.3 trillion dollars. It employs more than 300 million people worldwide [1] and generates more than 2% of the planet’s GDP contributing to economies all over the world [2]. However, simultaneously fashion industry has been criticised for having a negative impact on environment and society, i.e., increased CO2 emissions, high water consumption, harmful chemicals, textile waste, and poor working conditions (unsafe working environments and low salaries) [3–6]. For instance, textile industry produces 8–10% of the world’s carbon emissions, excluding the retail and consumer emissions from transportation and laundering [7]. Moreover, fashion industry is responsible for over 92 million tonnes of waste produced per year and 79 trillion litres of water consumed [8]. Additionally, the use of child labour, long working hours and chemically polluted production has a negative effect on the health of employees and communities living next to manufacturing locations [9]. Turning to Europe, textile consumption causes on average the fourth highest pressure on the environment and climate, following the consumption of food, housing, and mobility [10]. Nevertheless, despite these negative consequences for climate and people, fashion industry continues to grow [2] and such growth is largely explained by fast fashion and “buy-and-throw-away philosophy” when low costs of production and low prices create a culture where individuals are encouraged to do three things: to buy more, to buy more frequently and wear items for less time [11,12].

To address overconsumption and accordingly harm on natural environment and society at large [13], recently, debates in the field of fashion industry are becoming increasingly related to “sustainability awareness” [14,15], assuming and concluding that sustainability has the power to change the fashion demand while driving consumers’ purchasing decisions [4]. As consumer interest in sustainability constantly grows [13], this motivates companies to change their business models from traditional to [16,17] sustainability-reflecting models considering three “prices” paid in the fashion industry, namely the economic, social, and natural “price” [18].

In broad terms, sustainable fashion refers to “clothing and behaviours that are in some way less damaging to people and/or the planet” [17]. Sustainable fashion displays the challenge to fashion industry producers, retailers and other stakeholder of the supply chain by suggesting that “fast fashion needs to slow down” [19].

Previous literature in the field of sustainable fashion consumption has generally focused on several areas: first, on establishing an academic understanding towards sustainable fashion [18]; second, on analysing consumer attitudes and purchasing behaviour, mainly discussing attitude-behaviour gap [20], and third, on exploring the sustainable fashion consumption among various demographic groups of consumers [5,9,20,21]. This notwithstanding, acknowledging the gender-based difference in sustainable fashion consumption the previous literature argues for the need to explore sustainable fashion consumption from the perspective of female consumers.

The call to analyse female consumers refers to previous research, which has demonstrated that female consumers tend to buy more clothing than male [20]. Still, the female consumer group is also diverse in terms of age and social status. Previous studies focused on older female consumers’ sustainable fashion consumption [22], eco-conscious women in Chile [5], generation Y and Z female consumers in the UK [3], generation Z of both genders while later analysing the results separately [23], or young (18–25‐year olds) fashion‐engaged New Zealand females [24]. One of the conclusions from mentioned studies suggests that the cohort of women might matter when it comes to the way of thinking and acting, and consumption patterns [24,25]. In some cases, young women are even treated as key agents in fashion consumption because they are not afraid or ashamed to apply various forms of sustainable consumption, such as wearing second-hand clothing [26]. Thus, considering potential differences in female sustainable consumption based on their age, the paper focuses on two groups of women, namely students and university teachers.

The aim of the paper is to reveal the sustainable fashion consumption among female consumers while comparing two clusters of women: university teachers and students

More specifically, the paper seeks to answer the following research questions:

RQ1. What do women associate with fast fashion? Are there any differences between perceptions of two clusters: university teachers and students?
RQ2. How do women perceive sustainable fashion? Are there any differences between perceptions of two clusters: university teachers and students?
RQ3. What are the main general fashion consumption practices, what are sustainable consumption practices of females and do they differ for university teachers and students?
RQ4. What are the main drivers for sustainable consumption? Do they differ for university teachers and students?
RQ5. What are the main inhibitors for sustainable consumption? Do they differ for university teachers and students?

To answer the research questions, semi-structured interviews were conducted with 24 women, out of them 12 representing university teachers and 12 belonging to the student group.

The paper contributes to the scientific literature in several ways. First, consumers’ knowledge about sustainable fashion plays a significant role in shaping behaviour patterns [27]. However, the understanding of sustainable fashion consumption is still scant and needs some enhancement [28]. Accordingly, the paper broadens the understanding about consumption practices and main drivers and inhibitors for such consumption [29]. Second, following the previous notion that country of residence shapes the decision-making regarding sustainable consumption [30], this research focuses on Lithuanian consumers. Third, among demographic attributes, gender has been recognized as the most significant factor influencing consumer shopping [29] Differences in women’s and men’s consumption are often linked with gender roles and associated practices [31]. For instance, women’s taking care of household and other family members translates into behaviour when they purchase more food, clothing, and items for home than men do [29]. Thus, women purchase clothes not only for themselves, but also for others [32]. As such, women’s engagement in more sustainable consumption behaviour is really important.

[33] The current paper adds value to the current knowledge by analysing the attitude-behaviour gap regarding sustainable fashion consumption demonstrated by females [21]. Finally, the paper is based on comparison, as it seeks to reveal the differences between two social groups, namely university teachers and students.

The paper is organised as follows. The literature review deals with fast fashion, sustainable fashion, and sustainable fashion consumption concepts. Further, the methodology is presented. Next, the results are provided and after, comes the discussion part, which includes practical implications and research limitations. Finally, the conclusions end the paper.

## Literature review

### From fast fashion to sustainable fashion

Although the mass production of garments has existed since the 19th century, the fashion industry has significantly evolved over the past 30–40 years as a result of globalisation and liberalisation of the markets [8,12,34]. Due to the expansion of the industry boundaries [32], a new business model known as “fast fashion” started booming [35]. The New York Times used the expression “fast fashion” for the first time at the end of 1989, when Zara opened a shop in New York [4]. Generally speaking, fast industry refers to production of cheap items inspired by catwalk styles for quick sales in the mass retail market [36]. Accordingly, fast fashion is characterised by several attributes. First – by fashionable clothes. Fast fashion allows everyone to dress in accordance with the latest trends as clothing collections imitate current luxury fashion trends [34,36,37]. Consumers, especially females, engage in appearance management every day, which leads to buying appearance-related products, such as fast fashion items and by doing this they lower appearance-related anxiety [38]. Low price is the second characteristic. Mass-production, cost saving due to low quality or raw material and outsourcing the manufacturing to low labour cost countries enable offering cheap items to the customers [20,34]. The third characteristic refers to a speedy turnaround.

As rapid pace and cheap prices of fast fashion come at the cost not only to environment but also to labour force [39], and at the same time consumers are becoming more demanding in terms of sustainability across the entire value chain [4,40], sustainable fashion is growing in importance globally [41,42]. Sustainability itself is defined by approaches that address three fundamental aspects – social, economic, and environmental considerations [16]. However, the literature is not consistent and homogeneous when linking sustainability and fashion [43–45] and the terms such as eco-fashion, ethical fashion, green fashion, slow fashion or sustainable fashion are by some authors used interchangeably, while others make a clear distinction [9]. At the first glance, sustainability and fashion are two concepts, which inherently contradict each other [28] as fashion refers to short product life cycle [46], while sustainability is about durability. However, the scientific literature and business practices demonstrated by fashion houses, retailers or even fashion and lifestyle magazines provide plenty of examples how sustainability shakes and changes the fashion industry dominated by fast fashion [15,28].

From the general point of view, sustainable fashion refers to “apparel which incorporates one or more aspects of social and environmental sustainability” [5]. Wei and Jung (2017) [43] defined sustainable fashion as fashion items that either benefit or have no detrimental impact on our environment and society during their production and consumption, while also contributing to the creation of a sustainable future for humanity. Following this definition, fashion products produced with environmentally-friendly chemicals, using fabrics such as organic cotton or recycled materials, labour without exploitation, fair trade or products made for long-term use, all can be deemed sustainable fashion [43,47]. More recently, Mukendi et al. (2020) [17] described sustainable fashion as “the variety of means by which a fashion item or behaviour could be perceived to be more sustainable, including (but not limited to) environmental, social, slow fashion, reuse, recycling, cruelty-free and anticonsumption and production practices” (p.2874).

Sustainable fashion demands that sustainability should be addressed in several phases, namely the garment production-chain (from raw material to distribution); use of garments (consumer behaviour in terms of laundering and reuse); and post-consumer life (disposal) [5].

### Sustainable fashion consumption

According to previous studies, several ways of sustainable fashion consumption are known and applied [5,28,48]. It could include the purchasing of sustainable brands with environmentally friendly product design, adoption of organic fibres and raw materials, or use of recyclable packaging [49]. The other practice refers to buying second-hand clothing, which traditionally was deemed as being used primarily by consumers with modest financial means [50]. Nonetheless, the literature provides evidence that the motivation to go from second-hand shops has widened and also includes the desire to help the environment and to seek the authenticity of a vintage look in order to construct individuality and uniqueness of personality [51], and desire to have great-quality vintage garments or to address such practical considerations that children’s clothes are quite expensive and they grow quickly [52].

One more way of sustainable fashion consumption refers to swapping garments, which illustrates second-hand clothing consumption opportunities and is a part of sharing economy [53]. Swapping garments is a type of collaborative consumption, which is defined as “people coordinating the acquisition and distribution of a resource for a fee or other compensation” [54]. As such, items are exchanged on a “like-for-like” basis asking or getting some monetary remuneration [53]. Swapping, including swapping among family members and friends or neighbourhood, illustrates the emergence of conscious consumer behaviour and has been driven by several factors, such as environmental benefit, monetary benefits, moral responsibility or individual personality traits [53,55].

Another way of sustainable fashion consumption refers to the reusing of old clothes (the reuse occurs when a garment is used again) [56], re-purposing, which implies redesigning the clothes one already has or recycling [5,57]. Such practices again contribute to mitigating the negative impact fashion the industry makes globally.

Although consumers have a positive attitude towards environment and show the care about unethical behaviour, in many cases, this attitude does not translate into actions, which is known as attitude-behaviour gap [9,12,20,58,59]. This gap might be explained by different factors, also called the inhibitors for sustainable fashion consumption. The previous literature identified two types of barriers, namely internal and external [2,5,6,60]. Internal barriers relate to consumers themselves and include a lack of concern for the environment, limited knowledge about fashion consumption’s impact on people and environment, distrust or greenwashing of fashion companies, and demographic characteristics [6,60]. Other barriers which might also be defined as internal refer to motivation, values, locus of control, and perceived time and effort [6]. Concerning external barriers, which are independent of consumers, obsolescence pressures created by the fashion industry, limited range of sustainable clothing on offer, high price or social expectations could be mentioned. Moreover, barriers can be observed at several levels, namely at an individual level, at a social and cultural level and within the fashion industry [60]. Finally, it seems that barriers are mainly about accessibility, availability and affordability [2].

Moving towards a more sustainable life style, it is worth to analyse not only what hinders the sustainable fashion consumption, but also what encourages it. Turning to drivers, the recent research found that concern for the negative impact; good feeling while contributing to a life in a better world; being authentic and supporting local businesses and among the core drivers [5].

### Women’s fashion consumption

The previous literature supports the difference between male and female fashion sustainability awareness and accordingly consumption [20]. The study of Koca and Koc (2016) [61] found that male and female consumers had different perceptions and preferences regarding fast fashion and that women were more influenced by fashion while men were more influenced by brand name. Sun et al. (2020) [62] revealed that women tended to accept fast fashion low quality products more than men. More recently, Kopplin and Rösch (2021) [63] found that while women were inclined to buy sustainable clothing solely for environmental concerns, men either sought a way to express their identity or attain prestige through visible products. Overall, it was found that women were more likely to engage in sustainable consumption practices including purchasing organic food, using reusable bags or sustainable household products than men [31]. It was observed that the consumption patterns of women who belonged to the millennial age group focused on adopting sustainable fashion practices, including purchasing clothing from second-hand stores and vintage shops, engaging in renting, exchanging clothing, purchasing home-made clothing items, and exploring unique fashion [25]. Additionally, they also expressed interest in labour, social and environmental issues caused by the fashion industry [25]. It is interesting to mention that among women the primary factors influencing vintage consumption included fashion engagement, nostalgia proneness, and a desire for uniqueness. Conversely, second-hand consumption was primarily motivated by the sense of thriftiness [64].

Although it can be seen that women are more likely to engage in sustainable fashion consumption, it is important to consider their approach to fast fashion. One reason why women tend to choose the fast fashion alternative is the social media influence, accessibility, and the possibility to shop online. Research suggests that women were noted to exhibit a greater engagement in the consumption of fast fashion on social media platforms such as Instagram compared to men [64]. There are limited choices available to women in their purchasing habits, because sustainable fashion is more expensive and women tend to have a restricted understanding of it [65].

Nonetheless, women belonging to different age groups might differ in terms of their consumption habits and preferences of fast or sustainable fashion. The literature argues that young females (18–25 years old) are to be one of the most fashion-conscious consumer segments, understanding and following trends at a rapid pace and, not surprisingly, the biggest purchasers of fast fashion [66]. Thus, young female consumers are flexible, price-conscious and aware of trends [24]. 16–25‐year old consumers are trend‐based and tend to consume at the lower price end of fashion. Consumers over 25, on the contrary, are more likely to fit the ethically conscious consumer mould [24]. The current paper will provide insights into differences/similarities based on belonging to different age groups.

## Materials and methods

The Material and methods section describes the research context, data collection process, and data analysis process.

### Context.

In 2019, the textiles, clothing and leather industries in Lithuania accounted for about 1.5% of GDP. More recently, at the beginning of 2021, more than 900 enterprises (98% of them being small and medium sized) with around 24 thousand employees were active in the industry [67]. Fashion industry in Lithuania has centuries-old traditions, but recently the transformation towards sustainable production and consumption changes has been obvious [68]. Referring to production, Utenos Trikotažas serves as a brilliant example. It is the first textile producer in the world to fully comply with the Greenpeace environmental standards [69]. The company also implements various sustainability initiatives, such as zero-waste philosophy, 100% green energy, no toxic substances in production, focus on natural, organic or recycled fibres, and production only to order [69]. When it comes to consumption, a Lithuanian tech company Vinted deserves a changemaker title, as it is Europe’s leading online peer-to-peer second-hand fashion platform with more than 100 million members across 20 markets and around 800 million items listed on its sites in 2022 [70]. Turning to private consumer initiatives, clothing swaps or mending socks are becoming extremely popular in Lithuania [71,72].

### Data collection.

Having the aim of this paper in mind, qualitative research was used in the form of semi-structured interviews. Interview participants were recruited using purposive and snowball sampling methods. The research focused on female consumers, more specifically on university teachers and students in Lithuania. Twelve interviews with teachers and 12 interviews with students were conducted. The teachers and students were reached via personal contact of current paper co-authors.

In a research that employs semi-structured interviews, the sample size is often determined by the number of participants interviewed until “data saturation”, which refers to the point at which the data collection process no longer offers any new or relevant data, is achieved [73–75]. According to Hennink and Kaiser (2022) [74], data saturation can be achieved even with 9–17 interviews. In this research, data saturation was deemed to have been reached after 12 interviews with each group of participants.

Ethical considerations were respected throughout the research process. When making contacts with potential participants, information was provided about the aim of the research. Further, the voluntary nature of the participation in the research was highlighted. The consents were obtained from participant in verbal form. Next, the participants were informed about their right to quit at any time during the research process without any consequences. Later, during interviews, each interviewee was given sufficient time to share their experiences, opinion, and attitudes. Finally, the data were presented in such a way that no details that could be connected to a particular individual would be revealed. Thus, confidentiality and anonymity were guaranteed. All interviews were audio recorded and later transcribed. For each transcript/interviewee a code was given: the student transcripts were marked with codes S1, S2, etc.; the university teacher transcripts were marked with codes D1, D2, etc. While familiarising with data and generating initial codes, the translation of selected quotes from Lithuanian to English was made

In the interview guide, the following areas of focus were covered: perception of fast fashion and sustainable fashion; sustainable fashion consumption practices; the drivers and barriers of sustainable consumption. Interviews were conducted mainly face-to face in the Lithuanian language only. The duration of each interview was approximately 30 minutes.

Turing to informants’ profile, it could be mentioned that the average age of students was 21 years (age ranged from 20 to 24), whereas the average age of teachers was 45 years (age ranged from 31 to 59).

### Data analysis.

Analysis of the interview data was carried out in line with the six phases of qualitative thematic analysis proposed by Braun and Clarke (2006) [76]. The first phase involved the researchers familiarising themselves with the data while reading and re-reading the data several times. All 24 interviews transcripts were read and re-read by both researchers separately. During the second phase, initial codes were generated. Each researcher generated the codes independently. The third phase involved collating the codes into potential themes. The researchers tried to find the most appropriate theme based on codes. The next phase allowed to review the themes. At the fifth phase, the themes were defined and named. Finally, the research report was produced. All findings are supported by quotations. Both co-authors of this paper after working separately met to discuss their independent findings and agreed 100% on the final themes.

## Results

The results are provided according to the research questions.

RQ1. What do women associate with fast fashion? Are there any differences between perceptions of two clusters: university teachers and students?

The interviews results demonstrated that both groups, university teachers and students, were aware of the fast fashion phenomenon and used almost the same attributes to describe it.

Most participants mentioned that fast fashion associated for them with **poor clothing quality.** For instance, *“organisations, companies that produce such fashion products invest less in the quality of clothes, they focus more on the visual appeal” (*D10). Further, fast fashion was connected with **cheap garments or low price** arguing that “*the price is relatively not high”* (D2). However, one participant from the university teachers reflected that cheapness was just an illusion, as “*we would spend similarly if we bought more sustainably, but less*” (D1). Furthermore, **the promotion of overconsumption culture** was seen as part of fast fashion industry, as it: *“encourages the consumer to buy a lot at a relatively low price”* (D1). Actually, women revealed several aspects linked to the great excess of items. Initially, the quantity of items people buy are at the centre of fast fashion. Further, the periodicity dominates as fast fashion implies “*frequent purchase of clothes”* (D12). Next, such fashion is about growing and expanding the network of current consumers as companies try *“to sell these clothes < … > to the largest part of the population“* (S9). Finally, fast fashion was seen as an economic phenomenon with a long-term orientation which *”encourages further purchases” (*D8).

Going further, another attribute mentioned during the interviews referred to short life cycle of garments. It was noted that is *“it is a product for one season, you wear it, then next season you need another one“* (D9). One university teacher was really frank telling her story about the disposal of clothing after one season: “*I came, I bought it, I wore it for one season and then threw it away. The last time I did not even care if I washed it or not”* (D3). As such, basically, women contribute to the throw-away culture because of the psychological need to be fashionable as ”the fashion trend ends” (S4) or practical reasons as the clothes start “*showing wear quickly*” (D5).

Some of interviewees ascribed fast fashion to a certain customer age group or generation. For instance, one of the university teachers observed that fast fashion was oriented to *“younger people, youth”* (D11) and this position was supported by one student arguing that *“I think it is irrelevant for mature people or adults, because they do not really wear such clothes”* (S12).

One of the students associated fast fashion not with personal choice, but rather with the result of influence, as: *“it is a fast-changing, kind of a forced style, when everyone just likes the fashion of that period, then it changes again after a few months and everyone likes another thing again, so they keep buying newer and newer clothes”* (S3). Very similarly, another student related fast fashion with the actions of influencers: *“I think that fast fashion is a kind of fashion that comes at a certain time, for instance, from influencers or other people who are influential, and when we see how they dress, what clothes they choose on a daily basis, we renew our wardrobes and choose similar-style clothes”* (S7).

Some of the participants in both groups mentioned that fast fashion clothes were similar to designer clothes but less expensive, for instance, “*fast fashion is, well, as I like to say, cheap copies of expensive clothes*” (D4).

Another aspect that was observed in both groups was the association of fast fashion **with large brands and shopping centres.** For instance, *“fast fashion is something that you can find in shopping malls, for example, H&M, Primark or something, companies like that, where the clothes are probably made in China, on a large scale*” (S6) or *“Well, I think <…> stores like Zara, H&M, I think these are fast fashion stores, because the price is relatively not high”* (D2).

Looking at the sample, it was quite surprising that only a few participants raised **the concern and associated fast fashion with harmful production process**, empharising that fast fashion *“does not take into account how it is produced*” (D2) and accordingly *“no attention to environment”* (D2) is paid. Only several interviewees, more of them students, voiced the worry that fast fashion clothing was made unethically while exploiting workers: “*these clothes change very quickly and like the Shein brand, for example, are very cheap and it is not very clear how they are made or if people work in bad conditions and so on because you hear about it constantly; at least it reaches me through social media platforms like TikTok. These clothes change quickly and are cheap and somehow unethically made”* (S11).

Summing up, the participants consistently associated fast fashion with low quality products. They expressed concerns about materials and the disposable nature of fast fashion items. They were also concerned about the fast-changing nature, the trends coming and going quickly, it being a way to provide cheap clothing to a large portion of population. It encourages consumers to make frequent purchases, contributing to consumerism. Well-known brands such as H&M, Zara, Primark, and Shein were mentioned as examples of fast fashion. Some participants noted that fast fashion clothing resembled designer clothes but could be obtained at a significantly lower cost. While there is a general awareness of the environment impact of fast fashion, the emphasis was more on the rapid turnover of clothes than, for example, unethical production of fast fashion. Only several women, more of them students, raised questions about working conditions in the garment industry.

RQ2. How do women perceive sustainable fashion? Are there any differences between perceptions of two clusters: university teachers and students?

The research findings revealed that the participants from both groups agreed that sustainable fashion firstly referred to **good quality,** which is achieved because clothes *“are made of better material”* (S4). Another assigned feature implied the **longevity** dimension, which was perceived from two different perspectives, namely quality and circular economy. The first perspective linked longevity with quality as *“these clothes are of a higher quality and possibly intended for longer use”* (D6) and accordingly a person can enjoy the item longer seeing that “*those clothes don’t start showing wear so fast”* (D4). The second perspective underlined more the prolonging of the garment wear period when owners of the items change. The following quote captures both good quality and longevity and actually reflects the essence of the construct: *“Sustainable fashion… I think that first, the materials from which they are made are perhaps more organic or linen or something less synthetic and it is perhaps better in terms of wear, it is long lasting, those clothes do not wear out so quickly or something like that. Maybe so that it can be passed down from generation to generation, or, for example, from an older child to a younger one and so on”* (D4).

Coming back to the longevity perception, the second one is linked to the circular economy concept. It should be admitted that the majority of participants from both groups agreed that sustainable clothes could be or were fixed, exchanged, recycled, bought second-hand and this means that they **connect sustainable fashion with circularity.** For instance, *“sustainable fashion, I believe, is about buying used clothes from thrift stores, maybe exchanging with friends if it is available to you”* (S2) or *“people use clothes sustainably and try not to buy <…>, because the colour has already changed and to do that sustainably, that means re-sewing, buying second hand, sharing, in other words, using as little as possible and using as sustainably as possible”* (D9).

Some participants from both groups mentioned that sustainable fashion did not promote unethical production of clothes and worker exploitation: *“if we look at it from the user’s perspective, so that the workers who work in this field are not exploited, so that, let’s say, children do not work there or work there for half a dollar cent, as in the case of Sri Lanka”* (D1) or “*people who produce clothes in factories get much lower wages in fast fashion and in more sustainable fashion the wages are probably more acceptable”* (S6).

Moreover, several participants from both groups underlined that sustainable fashion was not as harmful to the environment and used eco-friendly materials. It is noteworthy that one of students associated sustainable fashion only with small business, *“where not so much is produced, but it is produced with quality”* (S11).

However, a couple of participants, namely from the university teachers’ group, did not believe that sustainable fashion existed or thought that it was impossible for it to exist. They were of the opinion that sustainability and fashion were mutually incompatible terms. The two quotes below illustrate their attitude.

“Sustainable fashion is an absolute nonsense to me, there is no such thing I think in life, as a woman. It is a myth, it is just it catches us emotionally, I know how much I was interested myself and how much I tried to be, and I wanted to be a part of it. I just do not really understand some things. I think that there is no such a thing. It is fiction. It is either fashion or it is not” (D3).

“Well, for me personally, sustainability and fashion are two absolutely incompatible words. Because sustainability is, well, fashion cannot be sustainable, I understand that there is such a term, but in my opinion, it is completely misleading, because if it is clothes that are sustainable, then they must have several aspects” (D11).

Summing up, the participants consistently associated sustainable fashion with good quality and longevity. It is perceived as clothing that can withstand extended use, possibly exchanged, repaired, recycled, or even passed down from one generation to another. It is also associated with the use of eco-friendly, organic materials; it is believed that sustainable fashion is less harmful to the environment and avoids unethical production practices. However, a couple of participants in the university teachers’ group expressed scepticism about the concept of sustainable fashion, felt that the term is misleading, or they were concerned about the fashion industry’s ability to achieve sustainability, because such clothes should meet multiple criteria.

RQ3. What are the main general fashion consumption practices, what are sustainable consumption practices of females and do they differ for university teachers and students?

Answering this research question, first, the results will be provided for university teachers, further for students and then the comparison will be done. Respectively, the general fashion consumption practices will be introduced first and sustainable consumption practices will come further.

The findings revealed several relevant points as regards teachers’ fashion consumption. First of all, for the majority, price and quality ratio seemed to be the core determinant when buying clothes. However, for some teachers, quality was much more important than price and accordingly: “*if there is no quality, I do not even look”* (D7). Additionally, individual feelings matter when purchasing, as it is important*: “so that I would like it and feel good with those clothes”* (D8). Next, the majority of teachers valued the items made by local producers, as they were *“really high-quality and very nice clothes”* (D2), even admitting that more expensive in comparison to acquiring clothes abroad or here in Lithuanian shops.

When turning to consumption, the teachers shared their different habits. Some of the teachers regularly made the inventory of wardrobe, for instance “*but I think that, well, let’s say, if I have not worn the clothes for two years in a row, it means that I do not need them”* (D1) and accordingly acted, namely: sell, reuse, swap, dispose, etc. The majority bought or at least tried to buy clothes when they really needed them. One frankly said that shopping for her was a “*leisure activity*” (D9), and people usually “*solve psychological problems with the clothes*” (D9). Going further, some of informants admitted than they specially purchased items abroad when going on conferences as it was less expensive; and such behaviour might be either spontaneous or deliberate. One of the teachers told than she regularly visited her favourite shops waiting for discounts. Incidentally, all informants admitted that discounts pushed them for more consumption. And finally, for the majority of teachers it was a regular practice to buy, at least for children, in fast fashion shops.

Turning to university teachers’ sustainable consumption practices, buying from second hand shops or Vinted platform, swapping, re-designing, re-purposing, buying with forethought and clothing recycling bins were mentioned. Further, these practices are explained in more detail.

Thus, it was found that more than a half of them at least once, mainly when they had been students, tried to buy fashion items in second hand shops. Moreover, some of them were still continuing doing this. However, the arguments behind choosing to purchase in second hand shops differed Some of females agreed that it was possible to find *“very high-quality and well-known name-brand clothes”* (D2), some enjoyed the diverse array of options available, as *“you can buy basically anything there”* (D6) or bought for particular occasions as *“some kind of performance, carnival, when new is difficult to find, and there are times when you need a certain colour or something”* (D11). Still, it seems that university teachers were quite sceptical about assigning purchases from second hand shops to sustainable fashion consumption, as *“Ponių Laimė [a second-hand shop] also promotes consumerism. When you buy that cheap piece of clothing, you really do not expect it to last long. <…> it is cheap, you can change it quickly, often*” (D10) and “*there is also a temptation, you buy a lot and unnecessary things there*” (D11).

The use of Vinted platform for selling and buying some items was also mentioned by a half of the university teachers: *“I think Vinted is also a very good example of sustainable fashion and I actually use it myself, especially when it comes to children, and I sell my own clothes” (*D1). Additionally, in some cases, the children of teachers encouraged them to use Vinted, as: *“my daughter has an account, so I also take part in Vinted to some extent”* (D6).

In line with the interview results, it seems that swapping, which also includes simply giving away or as a present, is the core sustainable fashion consumption practice mentioned by all 12 university teachers. Usually swapping occurs in the family or friends settings: *“I exchange clothes with my sister, with my daughter. I wear my son’s jacket”* (D3) or *“if I have not worn that item of clothing for a year and a half, then I will not wear it and I give it to my mother-in-law”* (D5). However, in separate cases, the clothes are given away to unfamiliar people who *“need help, <…> live in rural places”* (D11). Although it usually matters who becomes a new owner of items, as: *“I do not want to give it to just anyone”* (D4). One of the participants nonetheless admitted that non-used clothes is an issue and by swapping “*I transfer that problem to someone else*” (D11).

It is appealing that some items are being preserved and handed over in the families “*from generation to generation”* (D7), as for instance*: “I used to wear <…> my uncle’s jacket. <….> Both of my daughters wore it too and I am leaving it as an exhibit for my grandchildren”* (D7). The great quality of the material leads to garment durability, which in turn allows it to be used by multiple generations creating a perceived feeling of uniqueness as: “*I know that [the jacket] is unique*” (D7). Moreover, females are familiar with and participate in special swapping events for a particular stakeholder, usually mothers and children, or the whole small community living in a certain place: *“My eldership also organises that exchange once a year. All those circles that live by the principles of sustainability are notably the ones who do it”* (D9).

Some females noted that they use re-designing as practice for contributing to sustainable fashion consumption. For instance: *“I have reworked my husband’s sweater, because it was such a high-quality sweater, warm, woollen, so I just (adjusted) it, I know how to knit and sew a little. I scaled it back a bit and remade it for myself, and then I wore it”* (D6) or “*if it is a sweater or something that can be disassembled, I give it to my grandmother, and she then knits socks”* (D1).

Another frequently mentioned practice was about re-purposing of items, meaning that clothes continue their life-cycle but not for dressing purpose. The human garments are used for animals: *“we have a farm and a breeding kennel of Australian shepherds, so any sheets or blankets that lose their, as I say, commercial look, we always use them for the little puppies to keep them warm and well and so on”* (D1) or clothes *“end their lives as rags for cleaning”* (D8).

Next, referring to clothing recycling bins, some of the females reported that they used them quite frequently: *“in fact, I am very glad that we have a container called ‘give your clothes a second chance’ very close to home. <…>my clothes usually go into this container”* (D12). In the meantime, others told that they never had anything to put into the container because they always found another way to use their clothes.

Finally, buying with forethought can be introduced as one of the sustainable fashion consumption practices. Some of teachers admitted that in their youth or before children came, their purchases of clothes were more frequent and accordingly the wardrobe was full of items. However, this changed with age and children and “*now I try very hard to think about every purchase and think whether it can be combined with at least one other item and to not buy blindly*” (D1).

Turning to the students’ setting, it was revealed that some of them possessed a quite limited amount of garments while others had a lot – “*I have a lot of clothes”* (S6). Some of the students bought items *“to lift the mood”* (S4), one of them admitted even having a shopping addiction, as: *“I have such a problem that I like to shop”* (S5).

Eleven out of 12 students regularly and usually bought clothes in fast fashion shops such as H&M, Zara, Bershka, New Yorker, or Sinsay. Only one student noted that “*I have not been going to shopping malls and those H&Ms and Zaras for a long time*” (S6). The attitude to sustainability is not homogenous in the students’ group, as one student indicated that “*I try to buy clothes that are recycled. <…> to do as little harm as possible to the world”* (S5), while another, to the contrary, observed that *“unfortunately, I do not have a lot of money to pay attention to sustainability factors, so I do not notice them”* (S9).

Price and discounts seem to be the main aspects students pay attention to when buying garments; next, are the quality and comfort and one of students believed that “*if the item of clothing is comfortable, it is of good quality”* (S3). Even when trying to find *“the middle ground between price and quality”* (S4), the price generally won as “*clearly the price would be the deciding factor*” (S8) and accordingly “*I choose cheaper ones”* (S1). However, one student expressed the opinion that *“having one but of better quality, may be a little more expensive, but one quality item is better than hundreds of poor quality ones”* (S1).

It was surprising to discover that students did not prefer local producers, as *“I have not found such a manufacturer in Lithuania that I like”* (S2), or simply “*so far there has been no difference to me*” (S4).

Turning to students’ sustainable consumption practices, buying from second hands shops, Vinted platform, swapping, donating and clothing recycling bins were usually mentioned. Only one student referred to re-designing and re-purposing. Thus, buying from second-hand shops is a common practice influenced by the several factors, such as prices: “*I do not see the point in spending more money on clothes than they truly cost” (*D6) or quality, as in the second-hand shops there are “*better materials than in big stores”* (S12). Further, students were familiar with Vinted platform and bought and sold there quite often: “*I also try to buy clothes from Vinted <…> to support sustainable fashion”* (S5) or *“I had to sell it, but I really do not sell for cosmic money, <…> maybe it will be useful to someone, maybe someone will wear it, maybe it will suit someone since it did not suit me*” (S12). Next, swapping was mentioned by the students as well: “*I give some of my clothes to my younger sister”* (D5) or “*if I really do not wear it anymore, I put it in a bag. That bag rests for a couple of months and then maybe I have some guests, so I offer it, and so I give something away little by little”* (S6). Moreover, one student indicated donating as a sustainable fashion consumption practice: “*we have a family we know who was in a distress for some time, so we would give the clothes to them. They were very useful to them*” (S1). Going further, clothing recycling bins were mentioned quite often by the students, while only one student talk about re-designing: “*I ask my mother to make me shorts from jeans*” (S12) or re-purposing *“when a top is torn <…> I give it to my father to take to the garage for cleaning”* (S12).

Comparing sustainable fashion consumption findings among the two female groups, several aspects became obvious. First of all, the main finding from student interviews is quite saddening. While students expressed deep concern about environmental and even social impact of fast fashion industry, still they did not consistently apply such principles to their fashion consumption: 11 of 12 students mainly shopped in fast fashion shops driven by the lower price decision and hoping for some quality. Turning to teachers, the situation was somewhat different as less of them visited fast fashion shops and did so less frequently. Second, teachers had more knowledge and skills regarding fast or sustainable fashion and accordingly were more able to share their experience regarding consumption habits. It seems from the interviews that sustainable fashion is not the number one topic students discuss. Third, students did not value local producers, while for teachers, locally made clothes associated with quality. Fourth, swapping seemed to be the main sustainable fashion consumption practice for teachers, while students preferred second-hand shops. Fifth, teachers saw a lot of opportunities and value in garment re-designing and re-purposing, whereas students were barely familiar with these practices. Finally, in the case of students, swapping or buying from the second hand shops was mainly determined by the lack of money rather than sustainability considerations.

RQ4. What are the main drivers for sustainable consumption? Do they differ for university teachers and students?

Drawing upon the data of the interviews with teachers and students, several drivers for sustainable consumption were revealed: general awareness regarding fashion industry, social impact awareness, quality proposed by local producers, uniqueness and personal style, and item life-cycle extension. Nonetheless, these drivers were more indicated by teachers, as students mainly upheld the position that sustainability *“does not matter to me, as I want to buy as cheap as possible”* (S11).

The teachers mainly spoke about the awareness as a catalyst for behaving more sustainably. General awareness implies that *“it should be taken into account how the clothes are made and how much is consumed to make them and that billions of clothes are produced and how many more are thrown away”* (D2). It is vital to acknowledge that *“rubbish materials were used to make rubbish”* (D7). Such awareness explains the still rare is the situation when quality surpasses price as the primary determinant of purchasing: *“I am against certain [shops], well, for example, H&M, Zara, well, because it is really fast fashion for me and I do not buy these clothes, because they are not of good quality”* (D5).

Further, social impact awareness was indicated as a separate and extremely important driver. One of the teachers said that *“it is also important to me who works there and how much they earn or not. If those clothes cost a minimum, then those workers are probably exploited”* (D2). Although this concern was supported more by teachers, the students were more distant about this concern: only one of them mentioned that she tried, but it was difficult to find out “*where the item was made, <...>, who made it, was there any child abuse involved”* (S9).

Quality offered by the local producers serves as a driver for sustainable consumption only in case of teachers. As it was mentioned before, the approaches of teachers and students were essentially controversial. If the majority of teachers supported local producers buying items from them, the students did not prefer locally made garments at all. Teachers usually identified a local producer with quality, but complained about high price as “*unreasonable price*” (D5) is charged for the clothes. This notwithstanding, the local garment would win in both battles. Regarding the first fight, it was highlighted that “*if I had to choose between some fast fashion brand or a Lithuanian one and the latter would be more expensive, I would prefer the local one*” (D5). In terms of the second fight, the local item also won: “*If there were two very similar items that are very close in terms of their quality characteristics and the price, I would choose local, Lithuanian*” (D6). The two examples demonstrate that teachers give priority to local producers instead of cheap fast fashion items or foreign producers (not fast fashion) with similar characteristics and price.

Uniqueness and personal style were introduced as drivers for sustainable consumption by participants of both female groups. The students argued that *“you can create your own style by shopping second hand”* (S2) and *“those clothes are totally unique”* (S6). The teachers supported the notion that old clothes looked usually much better than fast fashion items and *“nobody can tell that they show signs of aging*” (S7).

Finally, for several participants from both groups, extension of the life-cycle seemed to be a reasonable driver for sustainable consumption: *“if you buy it not new, then you think that you are extending its validity, or to say, the time of being in the market”* (D6) or *“I did not wear it so <...> I sold it and maybe someone will use it, maybe someone will wear it”* (S12).

RQ5. What are the main inhibitors for sustainable consumption? Do they differ for university teachers and students?

Drawing upon the data of the interviews with teachers and students, several inhibitors for sustainable consumption were revealed, namely high price and low income, personal restrictions, constant advertising and invitation to buy, distrust of fashion brands, lack of information and knowledge, and limited social pressure.

The interviews allow for the conclusion that high price of garments and low income mainly hold the females back from sustainable fashion. One student explained that *“I do not have the income to afford something sustainable*” (S1) and this correlated with the situation of the teacher, who indicated that it was not affordable: *“you would make a huge dent in your budget by buying one pair of trousers”* (D5).

Turning to constant advertising and invitation to buy, it was reminded that the aim of each business is *“to produce and to sell”* (D8). The current situation is more than confusing and the discrepancy is obvious: *“the advertising does not work; one urges you to be more sustainable, the other – to buy more and more”* (D3). This raises the concern about the meaning of sustainable fashion in general, as one of the teachers observed: *“I do not agree that sustainable fashion is about buying second hand, because if it is frequent purchasing, it is no longer sustainable fashion”* (D12). Moreover, this concern led to the suggestion to reduce the availability of cheap items: *“so that as few of those unsustainable solutions remain on the market, not sold, not accepted, and if there were other solutions, they should be easy to use”* (D11). However, it should be admitted that students were also supporting the activities of designers, retailers or even on the state level to highlight the negative impact of fast fashion or even to regulate this market.

Personal restrictions imply several aspects. Firstly, bad experience with the Vinted platform was mentioned, as *“with Vinted <…> I was very disappointed, because those clothes have been there [not bought by anyone] for several years”* (D4). Further, some females preferred contact buying, as: *“I need to touch the item, I need to see it in person, try it on, and not to buy them right away”* (S2). Moreover, using Vinted was time consuming: *“I do not have time, <…> to chat and waste time for a few euros”* (D2). Buying in second hand shops required some patience and some females noted: “*I lack the patience to find what I like in those Humana shops”* (S3). Finally, it demanded some skills and some respondents conurred that *“I do not know how to shop there myself. Well, I cannot, I have a friend who likes to dawdle, and I cannot find such items as she does”* (D9).

Distrust of fashion brands was introduced as a barrier for sustainable fashion consumption. Cases were mentioned when companies declare commitment to sustainability, but *“send a bunch of plastic bags”* (D2). Moreover, one of the teachers even warned that *“if, for example, I heard that some company was socially irresponsible, I would definitely not buy from such a company”* (D1). The mentioned cases and provided quotations serve as signals of distrust in fashion brands as the behaviour of companies do not fit their declarations or message they convey to consumers.

Lack of information and knowledge were mentioned by teachers and students among the most important inhibitors for sustainable fashion consumption. The need for “*social advertising”* (D6) or a real example showing “*what happens because of those dies”* (D8), having in mind the vast quantity of water consumed in the process, were suggested. Such examples could lead to re-arranging the wardrobes.

Not fashionable clothes were also included in the list of barriers. As one research participant noted: *“these barriers are probably in the clothes themselves, some of which may not be so fashionable, <…> as you say, stylish”* (D4).

Limited social pressure as a barrier was mainly mentioned by students. One of the students shared her position: *“Since there are not many people among my friends who would advocate this, it is like no one has any influence on me in this matter. But if, I think, there would be, say, a friend who would really promote that sustainable fashion, I think that I would also start promoting it and just try not to buy unnecessary clothes”* (S5).

## Discussion

The current paper aims at revealing sustainable fashion consumption inherent to females, specifically university teachers and students. The core findings revealed the inconsistency between attitude and behaviour: although females were aware of sustainable fashion elements, at the same time, they (especially students) did not consider sustainability as a primary motivation for their behaviour. The representatives from both clusters shopped at fast fashion retailers’; however, students did it significantly more frequently and intensively in comparison to teachers. The teachers generally tended to choose swapping and redesigning as sustainable fashion consumption practices. Students predominantly mentioned second-hand shops and Vinted. Drivers leading to sustainable consumption included awareness, uniqueness and personal style, and item life-cycle extension. Speaking of the barriers, high price and low income, personal restrictions, and lack of information and knowledge were mainly noted.

According to interview data, it seems that both university teachers and students have knowledge about fashion industry and without difficulty can discuss the negative impact of fast fashion while encouraging themselves and others to select sustainable clothing alternatives. However, when it comes to recognising sustainability in fashion industry, it is obvious that for both groups it is in not easy to make a distinction which company belongs to fast fashion, which company, albeit still being a fast fashion brand, takes some steps towards sustainability, or which company truly commits to sustainability. An especially huge gap between attitude and behaviour, known also as the Fashion Paradox [12], was observed in the case of students, as almost all of them regularly and quiet often bought from fast fashion retailers. Such findings correspond to the previous research demonstrating that consumers are aware what sustainable fashion encompasses.However, these factors seemingly do not act as key determinants in the final decision-making process [9,21]. Accordingly, this contradicts the general belief expressed within academia that “younger generations are the most engaged in sustainable issues” [12], but this is in line with the research of Park and Lin (2020) [57] where albeit showing the highest interest in ethical fashion, young consumers were the ones actually buying less of it. Although this situation could be explained by low economic power of young people, the attitude-behaviour gap still needs to receive more attention from policy makers, and sustainability activists, otherwise the discrepancy between attitude and real behaviour will grow and sustainability consciousness will not thrive. Moreover, there is the need to clearly emphasise that sustainable fashion is affordable not only for consumers with high income, although the current study and previous research which was done among females in Chile [5] have revealed that females usually associate sustainability with high price. Accordingly, high price means that after buying a sustainably made item of clothing, less money is left for other demands or needs.

Turning to sustainable fashion consumption practices, swapping, buying from second-hand shops or online platform Vinted were mainly mentioned by the current research participants. These practices belong to common sustainability-reflecting practices identified among different gender and age groups in previous literature [5,28,48]. During interviews, it was underlined (and this links with previous studies) that the mentioned practices not only extended the useful life of items [52], but were also contributing to spending less [3]. Although Vinted has the potential to build the community of fashion-conscious individuals [20], university teachers and students pointed out that it was time-consuming. Such experience calls for re-thinking how to make sustainable consumption, including online, more user friendly despite the fact that generation Z is considered the first truly digital generation.

As regards second-hand shops, it is important to underline that they are not any more perceived as donation or place for poor people, but are related to four types of benefits in terms of sustainability, namely: economic value attained due to the low prices; hedonic value gained due to treasure hunting; uniqueness resulting from product attributes; and finally environmental value attained due to reducing consumption of new items [52]. Nonetheless, the core question which was raised by the participants of current research remains open – does frequent buying of unnecessary things from second hand shops because they are not expensive reflects the sustainability notion?

Going further to sustainable fashion consumption, the study among eco-conscious women in Chile found that re-designing and re-purposing were employed as the way to reduce the amount of disposed garments sent to landfills [5]. According to the current study, the mentioned practices are familiar to and often used by university teachers, but not so much by students. Such difference could be explained by the soviet heritage when possibility to buy fashionable clothes was limited and all females were able to sew and to knit in order to stand out from the crowd and express self-identity. Nowadays, young females are not in need to re-design the clothes as the availability of them is huge.

Regarding the drivers of sustainable fashion consumption, previous studies demonstrated that concern of the negative impact of fashion industry, supporting local producers, authenticity of sustainable fashion or good feeling while doing good things were important for women [5]. The current study found the same drivers. Looking at the differences between teachers and students, the obvious distinction was expressed regarding local producers. It seems that the young generation does not want to support the local manufacturers and workers as price for them is the core criterion. Such insights also dominate in the previous literature, especially referring to the generation Z consumers [21].

Turing to barriers preventing females from sustainable fashion consumption, the findings of the current study echo earlier insights, and as such, price and lack of information are prevailing.

The literature claims that sustainable fashion is more expensive than fast fashion [28]. Accordingly, it seems that many females, especially in young age, are unable to access sustainable fashion due to economic restrictions [3]. Hence, price contributes to the attitude-behaviour gap [3,77]. However, a study of young consumers in the UK revealed that they supported a tax for unsustainable fashion items [3]. Such support means that young people would be willing to pay even more with intention to improve the sustainability of the industry. During the interviews of this study it was also mentioned that some regulations might lower the desire of companies and consumers to behave in a unsustainable manner. Regardless, the price difference between sustainable and fast fashion items was indicated as a core factor when making the decision.

Lack of knowledge and information was as the red line in all the 24 interviews conducted with female. Teachers and students were more than honest explicitly acknowledging that they lacked knowledge and information about how to change their habits in favour of sustainable fashion consumption. The importance of information spread by retailers, sustainability enthusiasts, government, educational institutions, social media, or the influencers has been actually underlined in previous research [4,20]. Recommendations, leading by example or simply discussions among students and teachers at university might serve as determinants for increasing both groups’ sustainability awareness, leading to a lower environmental footprint and long-term usage of fashion items. The role of social advertising should be taken into consideration, as emotional appeals in advertisements drawing on environmental consciousness can especially target the feelings of women increasing or decreasing their desire to buy or avoid buying fast fashion. Accordingly, higher level of knowledge could lead to consumers demanding more socially responsible behaviour from fashion companies.

Summing up, the current study supports the previous notion in the literature regarding the attitude-behaviour gap which is also applicable to women in Lithuania, especially students.

### Practical implications

The paper provides several practical implications, which mainly address the role of three stakeholders, namely education institution, fashion brands and sustainable fashion sellers, and the enthusiasts of sustainable fashion. Firstly, the role of education should not be underestimated in changing the lifestyles of females and strengthening the sustainable culture [12]. This is extremely relevant tackling the students as a group, because they are significant consumers of both fast fashion and online purchases [27]. Several good practices how to teach sustainability in fashion design courses [78] have been already presented in the literature. However, the universities should feel more responsible for incorporating sustainable fashion practices into the curriculum and provide informal training sessions for raising students’ awareness how to deal faced with two options: lower price or sustainable item. On the other hand, the university teachers should also receive support from their more experienced colleagues how to start or continue to change behaviour while addressing overconsumption issue.It seems that women are in a quite unusual and challenging situation. On the one hand, society is increasingly attempting to lead a more sustainable life style. Accordingly, the social pressure for fashion companies is increasing while demanding to implement and follow sustainable strategies and practices. However, on the other hand, women lack knowledge how to distinguish sustainable products and brands from non-sustainable ones. Sometimes, women even lack knowledge about the impact of their overconsumption. Here, the people who are deeply engaged with environmental and social problems caused by fashion industry might take the leading role in spreading knowledge and initiating behavioural changes. Social advertising might also play a significant role in furthering consumption changes due to its emotional impact, especially for females.

Secondly, fashion brands should be encouraged to be more transparent and provide comprehensive information on the product journey from raw material to final fashion item. Such information would help understanding the complexity and, as it was suggested in earlier literature, calculating the price of one item per year, assuming that in the long term, sustainable clothes become less expensive than fast fashion throw-away clothes [12].

Thirdly, the sustainable fashion producers and sellers do not always take into consideration the features of different genders or age groups. In order to encourage women to rethink consumption patterns, the use of sustainable consumption channels should be as simple as possible. For instance, second hand shops should be enjoyable for consumers and display the clothing clearly, not making it difficult to find them [21]. Online platform Vinted should be easy-to-use even for people older than generation Z. Moreover, small or family-run businesses, reflecting the circular economy idea, could offer the services of clothes re-designing for a reasonable price.

Finally, responding to the wish of the younger generation females to be encouraged by sustainable fashion enthusiasts or so-called influencers [4], the space for building a strong sustainable fashion influencers’ community should be made available. The consumers perceive influencers’ lifestyle as attractive and this makes it possible to impact their daily decisions leading to less consumerism and lowering the negative impact on environment and people.

### Limitations

The paper has several limitations which need to be addressed while designing and conducting further research. First, a comparative study between genders, namely males and females, could provide valuable insights into gender-based consumption patterns. Further, the research could incorporate income level as one of the main factors potentially affecting consumption habits. Next, as the study was conducted in one country, future research could be done in other countries to analyse the effect of culture and country economic development on sustainable fashion consumption. Finally, quantitative empirical data could contribute to investigation of purchasing intentions and the sets of factors driving fast or sustainable fashion consumption and accordingly promoting shifts in female mind-set from quantity to quality.

## Conclusions

The study aims to reveal the sustainable fashion consumption of females, particularly university teachers and students. It was discovered that women expressed the care for environment and people, which are negatively affected by the fashion industry. However, at the same time, females, especially students, did not consider sustainability as a primary motivation for their behaviour. Although teachers try to find more sustainable solutions, arguing that quality of garments is as important as or even more important than price, students prefer lower prices and accordingly buy from fast fashion retailers despite being disappointed with quality later. Reusability, repurposing as well as reselling or giving items to somebody else before disposal were revealed as important elements of sustainable consumption. Swapping seems to be the main sustainable fashion consumption practice of teachers, while students give priority to second-hand shops. It was interesting to find out that students did not appreciate local producers, while for teachers, locally made clothes associated with quality. Turning to drivers of sustainable consumption, general awareness regarding fashion industry, social impact awareness, quality offered by local producers, uniqueness and personal style, and item life-cycle extension were mentioned. As regards inhibitors for sustainable consumption, it seems that high price and low income, personal restrictions, constant advertising and invitation to buy, distrust of fashion brands, lack of information and knowledge, limited social pressure were mainly discussed. Summing up, the sustainable fashion consumption still needs to be promoted on different levels, especially in education, in order to firmly contribute to sustainable societies and SDGs implementation. Moreover, the social pressure on consumers could also be increased, encouraging sustainable fashion enthusiasts to share their stories or creating social campaigns and showing the reality of manufacturing or waste management processes in fast fashion industry. As such, sustainability literacy might contribute to increasing the consumers’ knowledge about overconsumption and enhancing their intentions towards sustainability-reflecting choices.

## References

[1] BOF, McKinsey&Company. The state of fashion report 2019 n.d [cited 2024 Feb 2]. Available from: https://www.businessoffashion.com/reports/luxury/the-state-of-fashion-2019/

[2] Papamichael I, Chatziparaskeva G, Voukkali I, Navarro Pedreno J, Jeguirim M, Zorpas AA. The perception of circular economy in the framework of fashion industry. Waste Manag Res. 2023;41(2):251–63. doi: 10.1177/0734242X221126435 36690647 PMC9983045

[3] Hur E, Faragher- Siddall E. Young consumer perspectives on government policy interventions for sustainable fashion consumption in the UK. Fashion Practice. 2022;14(3):405–27. doi: 10.1080/17569370.2022.2125149

[4] Gazzola P, Pavione E, Pezzetti R, Grechi D. Trends in the fashion industry. The perception of sustainability and circular economy: a gender/generation quantitative approach. Sustainability. 2020;12(7):2809. doi: 10.3390/su12072809

[5] Bianchi C, Gonzalez M. Exploring sustainable fashion consumption among eco-conscious women in Chile. Int Rev Retail Distrib Cons Res. 2021;31(4):375–92. doi: 10.1080/09593969.2021.1903529

[6] Harris F, Roby H, Dibb S. Sustainable clothing: challenges, barriers and interventions for encouraging more sustainable consumer behaviour. Int J Consumer Stud. 2015;40(3):309–18. doi: 10.1111/ijcs.12257

[7] Virta L, Räisänen R. Three futures scenarios of policy instruments for sustainable textile production and consumption as portrayed in the Finnish News Media. Sustainability. 2021;13(2):594. doi: 10.3390/su13020594

[8] Niinimäki K, Peters G, Dahlbo H, Perry P, Rissanen T, Gwilt A. The environmental price of fast fashion. Nat Rev Earth Environ. 2020;1(4):189–200. doi: 10.1038/s43017-020-0039-9

[9] Blazquez M, Henninger CE, Alexander B, Franquesa C. Consumers’ knowledge and intentions towards sustainability: a Spanish fashion perspective. Fashion Pract. 2019;12(1):34–54. doi: 10.1080/17569370.2019.1669326

[10] European Environmental Agency. Textiles in Europe’s circular economy. 2019.

[11] Anguelov N. The dirty side of the garment industry: Fast fashion and its negative impact on the environment and society. New York, NY: CRC Press; 2015.

[12] Blas Riesgo S, Lavanga M, Codina M. Drivers and barriers for sustainable fashion consumption in Spain: a comparison between sustainable and non-sustainable consumers. Int J Fashion Des Technol Educ. 2022;16(1):1–13. doi: 10.1080/17543266.2022.2089239

[13] Yan R-N, Diddi S, Bloodhart B. Predicting clothing disposal: the moderating roles of clothing sustainability knowledge and self-enhancement values. Clean Responsible Consumption. 2021;3:100029. doi: 10.1016/j.clrc.2021.100029

[14] Abbate S, Centobelli P, Cerchione R, Nadeem SP, Riccio E. Sustainability trends and gaps in the textile, apparel and fashion industries. Environ Dev Sustain. 2023:1–28. doi: 10.1007/s10668-022-02887-2 36788931 PMC9912224

[15] De Ponte C, Liscio MC, Sospiro P. State of the art on the Nexus between sustainability, fashion industry and sustainable business model. Sustainable Chem Pharm. 2023;32:100968. doi: 10.1016/j.scp.2023.100968

[16] Pereira L, Carvalho R, Dias Á, Costa R, António N. How does sustainability affect consumer choices in the fashion industry? Resources. 2021;10(4):38. doi: 10.3390/resources10040038

[17] Mukendi A, Davies I, Glozer S, McDonagh P. Sustainable fashion: current and future research directions. Eur J Mark. 2020;54(11):2873–909. doi: 10.1108/ejm-02-2019-0132

[18] Henninger CE, Alevizou PJ, Oates CJ. What is sustainable fashion? J Fashion Mark Manag Int J. 2016;20(4):400–16. doi: 10.1108/jfmm-07-2015-0052

[19] Dory K. Why fast fashion needs to slow Down 2018. [cited 2024 Feb 5]. Available from: https://www.unep.org/news-and-stories/blogpost/why-fast-fashion-needs-slow-down

[20] Hageman E, Kumar V, Duong L, Kumari A, McAuliffe E. Do fast fashion sustainable business strategies influence attitude, awareness and behaviours of female consumers? Bus Strat Env. 2023;33(2):1081–98. doi: 10.1002/bse.3545

[21] Williams A, Hodges N. Adolescent Generation Z and sustainable and responsible fashion consumption: exploring the value-action gap. Young Consumers. 2022;23(4):651–66. doi: 10.1108/yc-11-2021-1419

[22] Kim G, Jin BE. Older female consumers’ environmentally sustainable apparel consumption. J Fashion Mark Manag Int J. 2019;23(4):487–503. doi: 10.1108/jfmm-04-2019-0068

[23] Djafarova E, Bowes T. ‘Instagram made Me buy it’: generation Z impulse purchases in fashion industry. J Retail Cons Serv. 2021;59:102345. doi: 10.1016/j.jretconser.2020.102345

[24] McNeill L, Venter B. Identity, self‐concept and young women’s engagement with collaborative, sustainable fashion consumption models. Int J Consumer Studies. 2019;43(4):368–78. doi: 10.1111/ijcs.12516

[25] Araújo MKF de, Mesquita RF de, Matos FRN, Sobreira M do C. Fashion consumption practices of millennials women: between fast and slow fashion. Revista de Administração Da UFSM. 2022;15(4):615–33. doi: 10.5902/1983465970206

[26] Taylor M, White KM, Caughey L, Nutter A, Primus A. Unique and cheap or damaged and dirty? Young women’s attitudes and image perceptions about purchasing secondhand clothing. Sustainability. 2023;15(23):16470. doi: 10.3390/su152316470

[27] Busalim A, Fox G, Lynn T. Consumer behavior in sustainable fashion: a systematic literature review and future research agenda. Int J Consumer Studies. 2022;46(5):1804–28. doi: 10.1111/ijcs.12794

[28] Lundblad L, Davies IA. The values and motivations behind sustainable fashion consumption. J Cons Behav. 2015;15(2):149–62. doi: 10.1002/cb.1559

[29] Workman JE, Cho S. Gender, fashion consumer groups, and shopping orientation. Family Consum Sci Res J. 2012;40(3):267–83. doi: 10.1111/j.1552-3934.2011.02110.x

[30] Bucic T, Harris J, Arli D. Ethical consumers among the millennials: a cross-national study. J Bus Ethics. 2012;110(1):113–31. doi: 10.1007/s10551-011-1151-z

[31] Bloodhart B, Swim JK. Sustainability and consumption: what’s gender got to do with it? J Soc Issues. 2020;76(1):101–13. doi: 10.1111/josi.12370

[32] Djelic ML, Ainamo A. The coevolution of new organizational forms in the fashion industry: a historical and comparative study of France, Italy, and the United States. 1999;10:622–37.

[33] Rahaman MdA, Ali MdJ, Wafik HA, Mamoon ZR, Islam MdM. What factors do motivate employees at the workplace? Evidence from service organizations. J Asian Finance Econ Bus. 2020;7(12):515–21. doi: 10.13106/JAFEB.2020.VOL7.NO12.515

[34] Bhardwaj V, Fairhurst A. Fast fashion: response to changes in the fashion industry. Int Rev Retail Distrib Cons Res. 2010;20(1):165–73. doi: 10.1080/09593960903498300

[35] McNeill L, Moore R. Sustainable fashion consumption and the fast fashion conundrum: fashionable consumers and attitudes to sustainability in clothing choice. Int J Consumer Studies. 2015;39(3):212–22. doi: 10.1111/ijcs.12169

[36] Ozdamar Ertekin Z, Atik D. Sustainable markets. J Macromarketing. 2014;35(1):53–69. doi: 10.1177/0276146714535932

[37] Joy A, Sherry JF Jr, Venkatesh A, Wang J, Chan R. Fast fashion, sustainability, and the ethical appeal of luxury brands. Fashion Theory. 2012;16(3):273–95. doi: 10.2752/175174112x13340749707123

[38] Xiao Y, Liu M, Wu B. The effect of social appearance anxiety on the online impulse purchases of fashionable outfits among female college students during pandemic periods: the mediating role of self-control and the moderating role of subjective socioeconomic status. Psychol Res Behav Manag. 2023;16:303–18. doi: 10.2147/PRBM.S392414 36761413 PMC9904223

[39] Colucci M, Vecchi A. Close the loop: evidence on the implementation of the circular economy from the Italian fashion industry. Bus Strat Env. 2020;30(2):856–73. doi: 10.1002/bse.2658

[40] Dzhengiz T, Haukkala T, Sahimaa O. (Un)Sustainable transitions towards fast and ultra-fast fashion. Fash Text. 2023;10(1):19. doi: 10.1186/s40691-023-00337-9

[41] Dzhengiz T, Riandita A, Broström A. Configurations of sustainability‐oriented textile partnerships. Bus Strat Env. 2023;32(7):4392–412. doi: 10.1002/bse.3372

[42] Kim Y, Oh KW. Which consumer associations can build a sustainable fashion brand image? Evidence from fast fashion brands. Sustainability. 2020;12(5):1703. doi: 10.3390/su12051703

[43] Wei X, Jung S. Understanding Chinese consumers’ intention to purchase sustainable fashion products: the moderating role of face-saving orientation. Sustainability. 2017;9(9):1570. doi: 10.3390/su9091570

[44] Jung S, Jin B. A theoretical investigation of slow fashion: sustainable future of the apparel industry. Int J Consumer Studies. 2014;38(5):510–9. doi: 10.1111/ijcs.12127

[45] Tran K, Nguyen T, Tran Y, Nguyen A, Luu K, Nguyen Y. Eco-friendly fashion among generation Z: mixed-methods study on price value image, customer fulfillment, and pro-environmental behavior. PLoS One. 2022;17(8):e0272789. doi: 10.1371/journal.pone.0272789 35972928 PMC9380922

[46] Afonso Vieira V. An extended theoretical model of fashion clothing involvement. J Fashion Mark Manag Int J. 2009;13(2):179–200. doi: 10.1108/13612020910957707

[47] Shen D, Richards J, Liu F. Consumers’ awareness of sustainable fashion. Mark Manag J. 2013;23:134–47.

[48] Jägel T, Keeling K, Reppel A, Gruber T. Individual values and motivational complexities in ethical clothing consumption: a means-end approach. J Mark Manage. 2012;28(3–4):373–96. doi: 10.1080/0267257x.2012.659280

[49] Caniato F, Caridi M, Crippa L, Moretto A. Environmental sustainability in fashion supply chains: an exploratory case based research. Int J Prod Econ. 2012;135(2):659–70. doi: 10.1016/j.ijpe.2011.06.001

[50] Williams CC, Paddock C. The meanings of informal and second-hand retail channels: some evidence from Leicester. Int Rev Retail Distrib Cons Res. 2003;13(3):317–36. doi: 10.1080/0959396032000101372

[51] Yan R-N, Bae SY, Xu H. Second-hand clothing shopping among college students: the role of psychographic characteristics. Young Consumers. 2015;16(1):85–98. doi: 10.1108/yc-02-2014-00429

[52] Liang J, Xu Y. Second‐hand clothing consumption: a generational cohort analysis of the Chinese market. Int J Consumer Stud. 2017;42(1):120–30. doi: 10.1111/ijcs.12393

[53] Henninger CE, Bürklin N, Niinimäki K. The clothes swapping phenomenon – when consumers become suppliers. J Fashion Mark Manag Int J. 2019;23(3):327–44. doi: 10.1108/jfmm-04-2018-0057

[54] Belk R. You are what you can access: sharing and collaborative consumption online. J Bus Res. 2014;67(8):1595–600. doi: 10.1016/j.jbusres.2013.10.001

[55] Lang C, Joyner Armstrong CM. Collaborative consumption: the influence of fashion leadership, need for uniqueness, and materialism on female consumers’ adoption of clothing renting and swapping. Sustain Prod Consum 2018;13:37–47. doi: 10.1016/j.spc.2017.11.005

[56] Hedegård L, Gustafsson E, Paras MK. Management of sustainable fashion retail based on reuse– A struggle with multiple logics. Int Rev Retail Distrib Cons Res. 2019;30(3):311–30. doi: 10.1080/09593969.2019.1667855

[57] Park HJ, Lin LM. Exploring attitude–behavior gap in sustainable consumption: comparison of recycled and upcycled fashion products. J Bus Res. 2020;117:623–8. doi: 10.1016/j.jbusres.2018.08.025

[58] Siraj A, Taneja S, Zhu Y, Jiang H, Luthra S, Kumar A. Hey, did you see that label? It’s sustainable! Understanding the role of sustainable labelling in shaping sustainable purchase behaviour for sustainable development. Bus Strat Env. 2022;31(7):2820–38. doi: 10.1002/bse.3049

[59] Cairns HM, Ritch EL, Bereziat C. Think eco, be eco? The tension between attitudes and behaviours of millennial fashion consumers. Int J Consumer Studies. 2021;46(4):1262–77. doi: 10.1111/ijcs.12756

[60] Connell KYH. Internal and external barriers to eco‐conscious apparel acquisition. Int J Consumer Studies. 2010;34(3):279–86. doi: 10.1111/j.1470-6431.2010.00865.x

[61] Koca E, Koc F. A study of clothing purchasing behavior by gender with respect to fashion and brand awareness. Eur Sci J. 2016;12(7):234. doi: 10.19044/esj.2016.v12n7p234

[62] Sun Y, Cai HH, Su R, Shen Q. Advantage of low quality in short life cycle products. APJML. 2019;32(5):1038–54. doi: 10.1108/apjml-03-2019-0148

[63] Kopplin CS, Rösch SF. Equifinal causes of sustainable clothing purchase behavior: an fsQCA analysis among generation Y. J Retail Cons Serv. 2021;63:102692. doi: 10.1016/j.jretconser.2021.102692

[64] Cervellon M, Carey L, Harms T. Something old, something used. Int J Retail Distrib Manag. 2012;40(12):956–74. doi: 10.1108/09590551211274946

[65] Chang A. The impact of fast fashion on women. J Integr Res Reflect. 2020;3:16–24. doi: 10.15353/jirr.v3.1624

[66] Young Lee J, Halter H, Johnson KKP, Ju H. Investigating fashion disposition with young consumers. Young Consumers. 2013;14(1):67–78. doi: 10.1108/17473611311305494

[67] Innovation Agency Lithuania. Apparel, textile and leather industry 2022 [cited 2024 Feb 5]. Available from: https://inovacijuagentura.lt/business-sectors/apparel-textile-and-leather-industry.html?lang=en

[68] Bartkutė R, Streimikiene D, Kačerauskas T. Between fast and sustainable fashion: the attitude of young Lithuanian designers to the circular economy. Sustainability. 2023;15(13):9986. doi: 10.3390/su15139986

[69] Utenos Trikotažas. Sustainability and Certificates n.d. [cited 2024 Jan 31]. Available from: https://www.ut.lt/en/sustainability-and-certificates/

[70] More than a trend: how Lithuanian brands contribute to sustainability in fashion 2024. [cited 2024 Feb 5]. Available from: https://lithuania.lt/news/business-and-innovations-in-lithuania/more-than-a-trend-how-lithuanian-brands-contribute-to-sustainability-in-fashion/

[71] BNN Baltic News Network. Lithuanians on the way to choose sustainable fashion 2023 [cited 2024 Feb 5]. Available from: https://bnn-news.com/lithuanians-on-the-way-to-choose-sustainable-fashion-245901

[72] Malinauskaitė G. Clothes swaps and mending socks: Lithuanians embrace sustainable fashion 2023 [cited 2024 Feb 5]. Available from: https://www.lrt.lt/en/news-in-english/19/1992714/clothes-swaps-and-mending-socks-lithuanians-embrace-sustainable-fashion

[73] Guest G, Namey E, Chen M. A simple method to assess and report thematic saturation in qualitative research. PLoS One. 2020;15(5):e0232076. doi: 10.1371/journal.pone.0232076 32369511 PMC7200005

[74] Hennink M, Kaiser BN. Sample sizes for saturation in qualitative research: a systematic review of empirical tests. Soc Sci Med. 2022;292:114523. doi: 10.1016/j.socscimed.2021.114523 34785096

[75] Marshall B, Cardon P, Poddar A, Fontenot R. Does sample size matter in qualitative research? A review of qualitative interviews in is research. J Comput Inf Syst. 2013;54(1):11–22. doi: 10.1080/08874417.2013.11645667

[76] Braun V, Clarke V. Using thematic analysis in psychology. Qual Res Psychol. 2006;3(2):77–101. doi: 10.1191/1478088706qp063oa

[77] Wiederhold M, Martinez LF. Ethical consumer behaviour in Germany: the attitude‐behaviour gap in the green apparel industry. Int J Consumer Stud. 2018;42(4):419–29. doi: 10.1111/ijcs.12435

[78] Gam HJ, Banning J. Teaching sustainability in fashion design courses through a zero-waste design project. Clothing Textiles Rese J. 2020;38(3):151–65. doi: 10.1177/0887302x20906470

